# First Genome of the Brown Alga *Undaria pinnatifida*: Chromosome-Level Assembly Using PacBio and Hi-C Technologies

**DOI:** 10.3389/fgene.2020.00140

**Published:** 2020-02-28

**Authors:** Tifeng Shan, Jianbo Yuan, Li Su, Jing Li, Xiaofei Leng, Yan Zhang, Hongtao Gao, Shaojun Pang

**Affiliations:** ^1^ CAS Key Laboratory of Experimental Marine Biology, Institute of Oceanology, Chinese Academy of Sciences, Qingdao, China; ^2^ Center for Ocean Mega-Science, Chinese Academy of Sciences, Qingdao, China; ^3^ Laboratory for Marine Biology and Biotechnology, Qingdao National Laboratory for Marine Science and Technology, Qingdao, China; ^4^ Dalian Haibao Fishery Co., Ltd., Dalian, China

**Keywords:** wakame, kelp, Alariaceae, seaweed farming, genetic breeding, invasive species

## Introduction

The brown alga *Undaria pinnatifida* (Harvey) Suringar is an economically important kelp species native to the Northwest Pacific and has been extensively farmed as human food in East Asia for more than half a century (Yamanaka and Akiyama, 1993). It is also an important resource for extracting biologically active compounds such as fucoidans which have diverse applications in pharmaceutical and cosmetic industries (Zhao et al., 2018; Yoo et al., 2019). Its annual yield worldwide has been more than two million tons since 2012 (http://www.fao.org/fishery/species/2777/en). Nowadays *U. pinnatifida* has become a cosmopolitan species due to its worldwide spread in recent decades, attracting increasing public attention (South et al., 2017). It has been listed as one of the world's 100 worst invasive species (Lowe et al., 2000), and in Europe has been regarded as one of the top 10 worst invasive species (Gallardo, 2014).

As a member of Laminariales, *U. pinnatifida* has a life history involving the alternation between two heteromorphic stages, namely the macroscopic sporophyte and the microscopic gametophyte. The haploid gametophyte was preliminarily determined to possess 30 chromosomes (Yabu et al., 1988). Sexual reproduction occurs in the gametophytic phase, in which the eggs discharged by female gametophytes are fertilized by sperms released by male gametophytes. In addition to this major reproductive pattern, parthenogenesis and apogamy have long since been revealed to be important components of the life history (Fang and Dai, 1959; Fang et al., 1979; Nakahara, 1984; Shan et al., 2013). Recently, an unusual monoecious phenomenon has been observed in zoospore-derived gametophytes, which are able to form oogonia and antheridia simultaneously and give rise to sporophytes by selfing (Li et al., 2014; Li et al., 2017). The sporophytes can become mature and release zoospores. All these spores grow into male gametophytes at first, and monoecious phenomena will be observed in some of them under developmental conditions. This finding makes the life cycle of *U. pinntifida* more complicated than we have traditionally thought. On one hand novel breeding methods have been developed based on these findings (Shan et al., 2013; Li et al., 2017), and on the other hand the versatile reproductive ways are suggested to be beneficial for its worldwide spread. However, the molecular mechanisms underlying the various reproduction means remains unknown. Lack of genomic sequence information hinders such fundamental study in *U. pinnatifida*. Herein, we report for the first time the complete genome of a male gametophyte of *U. pinnatifida* at the chromosomal level.

### Value of the Data

The genomic sequence data can be used for genetic breeding applications, and elucidation of sex-determination and invasion mechanisms in *U. pinnatifida*. It has been the first reference genome of the family Alariaceae, which can be used in comparative genomics and evolutionary studies of Laminariales (kelp) species.

## Materials and Methods

### Sample Collection and DNA Extraction

**Figure 1:**
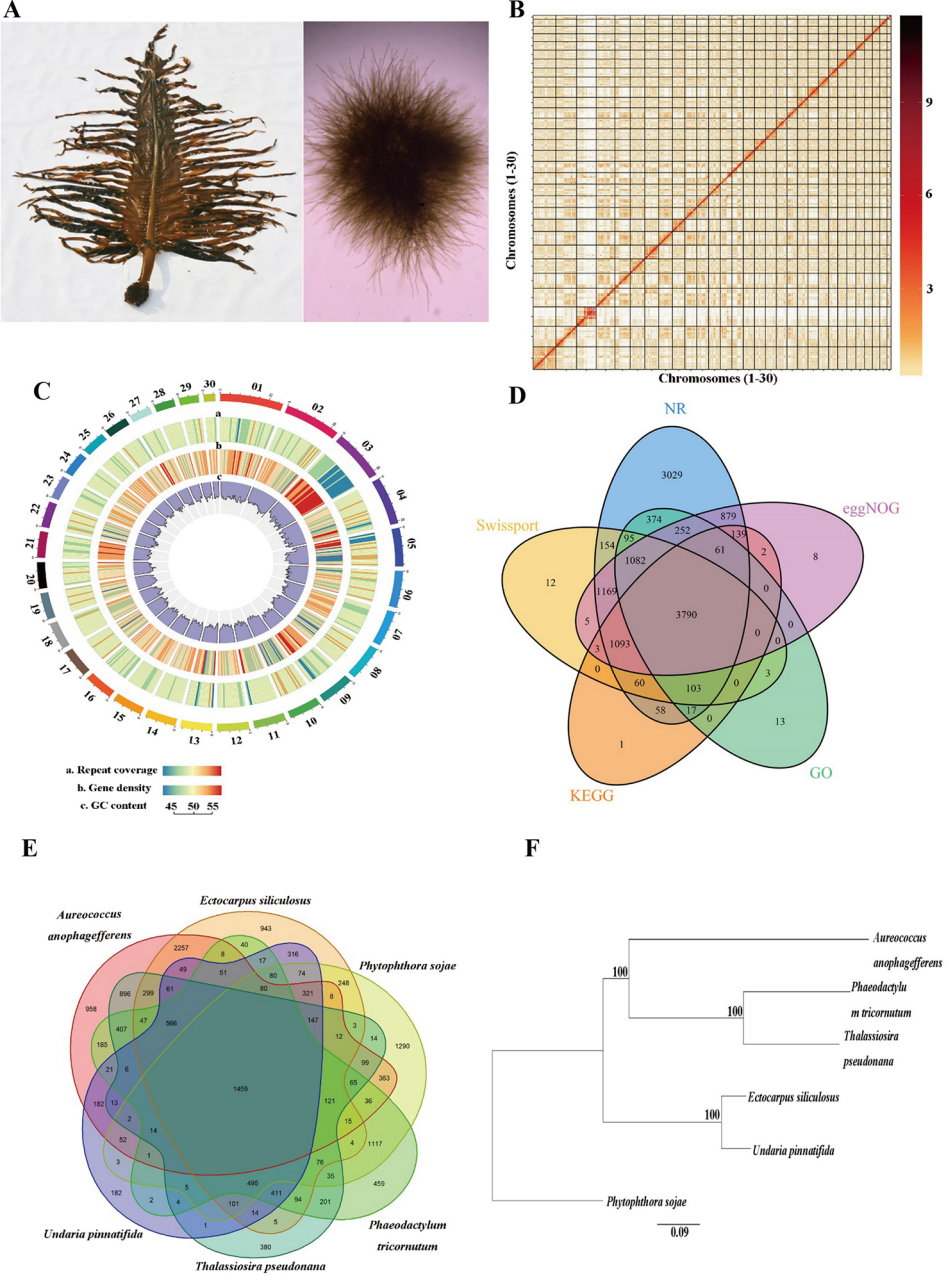
Genome assembly of *Undaria pinnatifida*. **(A)** Photo of sporophyte (left) and male gametophyte (right). **(B)** Hi-C interaction heatmap showing interactions among 30 chromosomes (the color bar on the right showing the interaction intensity). **(C)** Circos plot of 30 chromosome-level scaffolds (01–30) showing the genomic features. The color scales from the left (blue) to the right (red) show an increase in repeat coverage/gene density. **(D)** A Venn diagram showing the number of genes annotated in five databases. **(E)** A Venn diagram demonstrating orthologous gene families across six species. **(F)** Phylogenetic tree of the six species (bootstrap values higher than 50% were given).

One male gametophyte clone (designated as M23) of *U. pinnatifida*, which was established from one zoospore originating from a cultivated mature sporophyte (Pang et al., 2008) in Dalian, China, was used for genome sequencing (**Figure 1A**). Genomic DNA was extracted using the cetyl trimethyl ammonium bromide (CTAB) method according to Shan and Pang (2009). The DNA quantity and quality was assessed with Qubit 3.0 (Thermo Fisher Scientific Inc., Carlsbad, CA, USA) and agarose gel electrophoresis, respectively.

### Genome Survey

A survey of the genome was conducted using the Illumina sequencing. A short-insert DNA library (~280 bp) was established and sequenced by Illumina Novaseq6000 platform (Illumina Inc., San Diego, CA, USA). After discarding low-quality and redundant reads, we obtained 24.3 Gb high-quality paired-end (150 bp) clean reads (**Table 1**). These reads were employed in the distribution analysis of *k*-mer (*k*=19) frequency (Marçais and Kingsford, 2011). The peak of 19-mer was at a depth of 34X, and the genome size was predicted to be ~539 Mb. The heterozygosity and the percentage of repeated sequences were estimated to be 0.48% and 34.2%, respectively. Pilot assembly of the clean reads resulted in a genome of 551 Mb, similar to that estimated by *k*-mer method.

**Table 1:** Summary statistics of genome assembly of *Undaria pinnatifida*.

Sequencing platform	Clean data size (Gb)	Application
Illumina Novaseq6000 (short reads)	24.3	Genome survey and correction
PacBio Sequel (long reads)	62.3	Genome assembly
Illumina Novaseq6000 (Hi-C reads)	57.1	Assisted assembly at the chromosomal level
**Genome assembly and scaffolding at chromosomal level**		
Contig number	515	
Contig length (bp)	511,028,173	
GC%	50.14	
Contig N50 (bp)	1,707,374	
Scaffold number	114	
Scaffold N50 (bp)	16,510,065	
Scaffold length (bp)	511,280,173	
Chromosome length (bp)	502,827,406	
Hi-C mapping percent	98.4%	
**The predicted repeated sequences**		
Type	Number	Length (bp)
DNA	30,267	10,285,717 (2.0%)
LINE	62,383	17,880,936 (3.5%)
LTR	73,036	44,549,843 (8.7%)
RC	2,784	1,247,182 (0.2%)
SINE	326	17,815 (0.003%)
Unknown	785,477	184,368,999 (36.1%)
Low complexity	2,121	370,709 (0.07%)
Satellite	1,041	578,618 (0.1%)
Simple repeat	73,931	16,728,946 (3.3%)
Total	1,031,366	276,028,765 (54.0%)

LINE, long interspersed element; LTR, long terminal repeat; RC, rolling circle; SINE, short interspersed element.

### Long-Read Genome Sequencing With PacBio Technology

Genomic DNA was mechanically fragmented into sizes of ~20 kb using a Covaris g-tube (Covaris Inc., Woburn, MA, USA). The Pacific Biosciences single-molecule real-time (SMRT) Bell™ sequencing library was constructed using a Template Prep Kit (PacBio, Menlo Park, CA, USA). After DNA damage and end repair, SMRT adaptors were ligated to generate SMRT Bell™ templates. The Blue Pippin (Sage Science Inc., Beverly, MA, USA) was used to select sizes of the fragments (> 15 Kb). After a second round of DNA end repair, the SMRT Bell™ templates were purified for final sequencing with the PacBio Sequel system (PacBio, Menlo Park, CA, USA). Ten SMRT cells were used to obtain a total of 62.6 Gb (~120X) of raw polymerase reads.

### *De Novo* Genome Assembly

After removal of short and low-quality reads and the adaptor sequences, the raw polymerase reads were converted to 62.3 Gb subreads data, with an N50 length of 11,463 bp. Preliminary assembly was conducted using Falcon v1.2.4 (Chin et al., 2016). All the clean sequencing data was aligned to the assembled contigs with BLASR (Chaisson and Tesler, 2012), and errors in the contigs were corrected using Arrow (SMRT link v6). The Illumina sequencing data was aligned to the contigs using BWA v0.7.15 (Li and Durbin, 2009) for further correction by using Pilon v1.22 (Walker et al., 2014). A draft genome of 616.6 Mb which consisted of 807 contigs was obtained, with an N50 length of 1.8 Mb. The gametophytes of kelp species are known to contain symbiotic bacteria and thus a filtration procedure was conducted to remove potential bacterial contamination (Ye et al., 2015). The contigs were searched against the non-redundant nucleotide (NT) database of the National Center for Biotechnology Information (NCBI; https://www.ncbi.nlm.nih.gov/) with BLASTN and those with the best-hit matches to bacteria were discarded from the genome. The final draft genome was 511.0 Mb and consisted of 515 contigs with an N50 length of 1.71 Mb (**Table 1**).

### High-Throughput Chromosome Conformation Capture (Hi-C) Library Construction and Chromosome-Level Assembly

Gametophytic cells were fixed with formaldehyde (1.44%) and lysed with tissue lysis (40 mM CaCl_2_, 1 mg mL^−1^ collagenase). The cross-linked DNA was digested with the restriction enzyme DpnII. Biotinylated residues were added during repair of the sticky ends and the resulting blunt-end fragments were ligated under dilute conditions (Lieberman-Aiden et al., 2009). The DNA was extracted and randomly sheared to fragments of 250–500 bp. The biotin labeled fragments were isolated with magnetic beads, and end repair, dA tailing, adaptor ligation, PCR amplification, and purification were conducted for final construction of Hi-C library. The DNA quantity was preliminarily estimated by Qubit 3.0 and the insert size was tested by Agilent 2100 (Agilent Technologies, Santa Clara, CA, USA). The library concentration was accurately quantified by quantitative PCR. The qualified library was sequenced to produce 150 bp paired-end reads using Illumina Novaseq6000 platform. A total of 57.1 Gb clean dataset was obtained, containing 190,379,612 reads (**Table 1**).

The Hi-C sequence data was aligned against the draft genome using JUICER v1.6.2 (Durand et al., 2016). Totally 161,141,067 (84.6%) reads were mapped to the genome and 110,009,243 (57.8%) of them were uniquely mapped. The uniquely mapped sequences were analyzed with 3D-DNA software to assist genomic assembly (Dudchenko et al., 2017). The algorithms “misjoin” and “scaffolding” were used to remove the misjoins and obtain scaffolds at the chromosomal level. The algorithm “seal” was employed to find the scaffolds that had been incorrectly removed by the “misjoin”. The heatmap of chromosome interactions was constructed to visualize the contact intensity among chromosomes using JUICER v1.6.2 (**Figure 1B**). As a result 114 scaffolds were assembled with an N50 length of 16.5 Mb. Finally a total of 502.8 Mb genomic sequences were located on 30 chromosomes, accounting for 98.4% of the whole assembled length (**Table 1**).

### Genome Annotation

Tandem repeats and interspersed repeats were identified using TRF v407b (Benson, 1999) and RepeatModeler v1.0.11, respectively. RepeatMasker v4.0.7 was used to mask the predicted and known repeated sequences (Tarailo-Graovac and Chen, 2009) and RepeatProteinMask v4.0.7 was used to mask the known repeated protein sequences. Circos plot of 30 chromosome-level scaffolds (01–30) was constructed to show the genomic features (Krzywinski et al., 2009). Totally 276.0 Mb repeated sequences were masked, accounting for 54.0% of the whole genome length (**Table 1**, **Figure 1C**). We used tRNAscan-SE v1.4alpha (Lowe and Eddy, 1997) to predict tRNAs and identified other types of non-coding RNAs (ncRNA) through searching against the Rfam database v11.0 (Griffiths-Jones et al., 2005). A total of 507 ncRNAs were detected, including 367 tRNAs, 93 rRNAs, 43 sn RNAs, and 4 mi RNAs.

Protein-coding genes were predicted through the combination of homology-based, transcriptome-based, and *ab initio* predictions. The protein sequences of five related species, namely *Ectocarpus siliculosus*, *Thalassiosira pseudonana*, *Phaeodactylum tricornutum*, *Aureococcus anophagefferens,* and *Phytophthora sojae* were downloaded from NCBI and aligned against the assembled genome using GeMoMa v1.4.2 (Keilwagen et al., 2016). The full-length transcriptome, which was obtained from a pooled sample of gametophytes and sporophytes of *U. pinnatifida* in our previous study (unpublished data), were used to predict the open reading frames (ORFs) with PASA v2.0.2. For *ab initio* prediction, Augustus v3.2.2, GlimmerHMM v3.0.4 (Majoros et al., 2004), SNAP v1.0, and GeneMark-ES v4.33 (Besemer and Borodovsky, 2005) were used to predict the gene structure. All the prediction results were integrated with EVidenceModeler (EVM) (Haas et al., 2008), and the untranslated regions and alternative splicing were predicted with PASA. A total of 14,178 genes were predicted with an average length of 2,089 bp (**Figure 1C**).

Functional annotation of the predicted protein-coding genes were conducted through aligning them against the non-redundant protein (NR), SwissProt, evolutionary genealogy of genes: Non-supervised Orthologous Groups (eggNOG) (Huerta-Cepas et al., 2015) and the Kyoto Encyclopedia of Genes and Genomes (KEGG) databases using the BLASTX with an *E* value cutoff of 10^−5^. Annotation by the Gene Ontology (GO) database was performed using Blast2GO software. Totally 12,402 (87.4%) genes were annotated in at least one database (**Figure 1D**).

### Completeness and Accuracy of the Assembly

The previously obtained full-length transcripts was aligned against the assembled genome with Gmap and it was found 87.7% of the transcripts could be mapped.

The paired-end reads obtained in genome survey were also aligned against the assembled genome with BWA and 92.2% of them were successfully mapped. The analysis of Benchmarking Universal Single-Copy Orthologs (BUSCO) v3.0.2 (Simão et al., 2015), in combination with TBLASTN, Augustus, and HMMER v3.1b2 (Finn et al., 2011) software, was used to evaluate the completeness of the assembled genome based on eukaryota_odb9 database. The percentage of the identified complete BUSCOs was 82.9% at the protein level, with the fragmented and missing BUSCOs accounting for 8.9% and 8.2%, respectively.

### Ortholog and Phylogenetic Analysis

OrthoMCL v2.0.5 was used for ortholog analysis based on the protein datasets from *U. pinnatifida, Ectocarpus siliculosus, Phaeodactylum tricornutum, Thalassiosira pseudonana, Phytophthora sojae,* and *Aureococcus anophagefferens*. Totally 15,414 clusters were formed, and 874 of them were single-copy gene clusters (**Figure 1E**). The single-copy gene clusters were aligned using MAFFT v7.45 and the phylogenetic tree was constructed with maximum likelihood method using PhyML (http://www.atgc-montpellier.fr/phyml/binaries.php) (**Figure 1F**).

## Data Availability Statement

The raw genome sequencing data have been deposited in the NCBI SRA database under the BioProject accession number PRJNA575605. Genome assembly and annotation data has been deposited at Figshare (https://figshare.com/s/94aebbd77f374b9c6faf). The raw SMRT sequencing data for full-length transcriptome analysis is available in NCBI SRA database with accession numbers SRR8083207, SRR8083208, and SRR8083209.

## Author Contributions

SP and TS conceived the study. TS cultured and maintained the gametophyte samples. XL, YZ, and HG cultured the sporophytes. TS, JY, LS, and JL extracted the DNA and performed genome assembly and data analysis. TS, JY, and SP wrote the manuscript.

## Funding

This research was supported by the National Natural Science Foundation of China (No. 41676128), the Special Research Fund of Key Laboratory of Experimental Marine Biology, Chinese Academy of Sciences (No. KLEMB-SR01), the Biological Resources Program from Chinese Academy of Sciences (KFJ-BRP-017-27), China Agriculture Research System (CARS-50), the Taishan Scholar Program of Shandong Province, and the Foundation for Huiquan Scholar of Institute of Oceanology, Chinese Academy of Sciences.

## Conflict of Interest

Authors XL, YZ and HG were employed by company Dalian Haibao Fishery Co., Ltd., China.

The remaining authors declare that the research was conducted in the absence of any commercial or financial relationships that could be construed as a potential conflict of interest.
